# A scoping review of behavioural science approaches and frameworks for health protection and emergency response

**DOI:** 10.1177/17579139241257102

**Published:** 2024-06-10

**Authors:** Alice Zelenka Martin, D Weston, J M Kesten, C E French

**Affiliations:** MSc Public Health Student, University of Bristol, Canynge Hall, 39 Whatley Road, Bristol BS8 2PS, UK; NIHR Health Protection Research Unit in Behavioural Science and Evaluation at University of Bristol, Bristol, UK; Behavioural Science and Insights Unit, UK Health Security Agency, London, UK; NIHR Health Protection Research Unit in Emergency Preparedness & Response at King’s College London, London, UK; NIHR Health Protection Research Unit in Modelling and Health Economics at Imperial College London, London, UK; NIHR Health Protection Research Unit in Behavioural Science and Evaluation NIHR Applied Research Collaboration (ARC) West at University Hospitals Bristol and Weston NHS Foundation Trust, UK; Population Health Sciences, Bristol Medical School, University of Bristol, Bristol, UK; NIHR Health Protection Research Unit in Behavioural Science and Evaluation at University of Bristol, Bristol, UK; Population Health Sciences, Bristol Medical School, University of Bristol, Bristol, UK

**Keywords:** behavioural insights, emergency response, health protection

## Abstract

**Aims:**

Rapid intervention development, implementation, and evaluation are required for emergency public health contexts, such as the recent COVID-19 pandemic. A novel Agile Co-production and Evaluation (ACE) framework has been developed to assist this endeavour in future public health emergencies. This scoping review aimed to map available behavioural science resources that can be used to develop and evaluate public health guidance, messaging, and interventions in emergency contexts onto components of ACE: rapid development and implementation, co-production with patients or the public including seldom heard voices from diverse communities, and inclusion of evaluation.

**Methods:**

A scoping review methodology was used. Searches were run on MEDLINE, EMBASE, PsycINFO, and Google, with search terms covering emergency response and behavioural science. Articles published since 2014 and which discussed a framework or guidance for using behavioural science in response to a public health emergency were included. A narrative synthesis was conducted.

**Results:**

Seventeen records were included in the synthesis. The records covered a range of emergency contexts, the most frequent of which were COVID-19 (*n* = 7) and non-specific emergencies (*n* = 4). One record evaluated existing approaches, 6 proposed new approaches, and 10 described existing approaches. Commonly used approaches included the Behavioural Change Wheel; Capability, Opportunity, and Motivation Behaviour model; and social identity theory. Three records discuss co-production with the target audience and consideration of diverse populations. Four records incorporate rapid testing, evaluation, or validation methods. Six records state that their approaches are designed to be implemented rapidly. No records cover all components of ACE.

**Conclusion:**

We recommend that future research explores how to create guidance involving rapid implementation, co-production with patients or the public including seldom heard voices from diverse communities, and evaluation.

## Introduction

The role of health-protective human behaviour in combatting the spread of COVID-19 has highlighted the importance of behavioural science as a vital tool in the health protection and emergency response arsenal.^1^ Guidance for utilising behavioural science in routine public health is available.^2–4^ However, the challenge of developing and utilising behavioural science in a public health emergency and evaluating the implementation of interventions is that appropriate public health response behaviours need to be understood, and guidance must be formulated and disseminated rapidly to be implemented in time to prevent avoidable outcomes.^5,6^ It also needs to be responsive to evolving situations and able to be updated as the evidence base changes.^7,8^ For guidance to be effective, it needs to be delivered by a trusted source, consistent across all responding organisations, and credible and realistic.^5,6,8–10^ These standards can be challenging to meet under time pressure, especially when key behavioural science frameworks are typically designed for non-emergency health contexts.^4,11,12^ There is therefore a clear need to establish an evidence-based, effective framework for conducting rapid, emergency preparedness and response-focused guidance and intervention development, ahead of any future public health emergencies.

The Agile Co-production and Evaluation (ACE) framework aims to fulfil this need by supporting the rapid development and evaluation of public health interventions, messaging, and guidance involving co-production with target populations. Full details of the ACE framework, including how it was developed, its relevance to behavioural science theory, and how it could be utilised in practice, are explained in the study by Yardley et al.^13^ This framework focuses on (1) a need to rapidly develop and disseminate messages, guidance, or interventions; (2) consideration of how to involve and engage members of the public, especially under-served communities whose voices are seldom heard or incorporated into intervention development, and (3) use of rapid testing, evaluation, or validation methods (such as online testing or implementation evaluation using routine data) to provide feedback on effectiveness. These three points have been identified by the authors of this review (and co-authors of the ACE framework) as central to the approach; these criteria will be referred to hereafter as the ACE criteria.

Although the authors are unaware of any existing emergency preparedness and response-focused behavioural science framework that addresses all these constructs, this scoping review aimed to map the extent and type of available resources concerning behavioural science approaches and frameworks for health protection in emergency contexts. We wanted to gain insights into the extent to which existing approaches have been designed for rapid implementation and co-production with the target population. This will help to inform the ACE framework by leading to the development of recommendations for future work, with the aim of creating a framework for effective and timely implementation of interventions, guidance, and messaging as part of a public health emergency response.^13^

## Methods

The Joanna Briggs Institute (JBI) scoping review framework was used.^14^ A review protocol, based on the JBI^15^ template, is published on the Open Science Framework (DOI: 10.17605/OSF.IO/FE6TN).

The literature searches were conducted on MEDLINE, EMBASE, and PsycINFO on 16 June 2022 and on Google on 17 June 2022 by Alice Zelenka Martin. The search terms (Supplemental Material 1) covered two core topics: behavioural science and emergency response. We limited the search to records published since 2014; this was a pivotal year for the incorporation of behavioural science into emergency response^16^ and a crucial time in work to standardise and collate theories and approaches in behavioural science.^17,18^ A Google search captured grey literature, such as government and other public health-related documents (Supplemental Material 1). Only the first 10 pages of Google results ordered by relevance were screened.

The records, including the grey literature, were title and abstract, and full text screened in duplicate on Rayyan (https://www.rayyan.ai/) using our prespecified inclusion and exclusion criteria. Alice Zelenka Martin screened all records with duplicate screening of each record conducted by Dale Weston, Jo Kesten, and Clare French. Conflicting decisions between reviewers were resolved through a whole-group discussion.

Inclusion criteria:

1. Type of source: any type of record is eligible.
2. Subject: the record must address guidance or frameworks for utilising behavioural science in an emergency response context; this may include evaluations or assessments of existing guidance/frameworks, discussions on developing new guidance/frameworks, and discussions on the presentation of existing guidance/frameworks.
3. Context: behavioural science must be applied to an emergency of public health relevance. Records published or covering emergencies in any country are eligible.
4. Other: records published since 2014; English or with an English version available.

Alice Zelenka Martin performed data extraction using a predefined Excel database, and co-authors discussed the results. Data were extracted based on JBI guidance^14,15^ and the ACE criteria. Data were synthesised to identify: (1) who is producing guidance and frameworks; (2) emergency contexts; (3) co-production with patients or the public; (4) consideration of how to engage diverse populations; (5) to what extent approaches incorporate testing or evaluation; and (6) who approaches are being created for. Groupings 1 to 5 were prespecified, and grouping 6 developed iteratively as we synthesised the data. Results were narratively synthesised.

## Results

**Figure 1:**
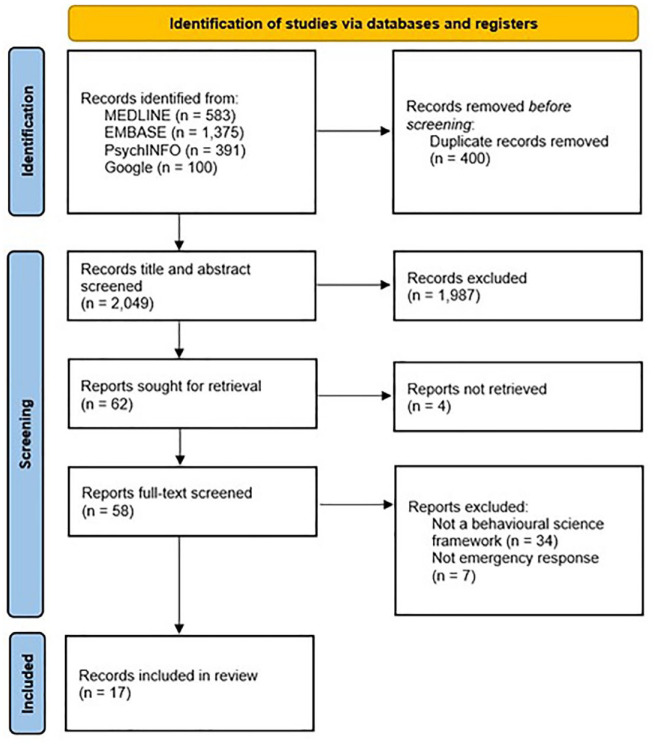
Preferred Reporting Items for Systematic Reviews and Meta-Analyses flow chart

The database and Google searches identified 2449 records. Following the removal of duplicates, 2049 records underwent title and abstract screening. Sixty-two records were sought for full-text screening, but four could not be retrieved. Seventeen records were included for data extraction (Figure 1).

### Characteristics of included articles

**Figure 2:**
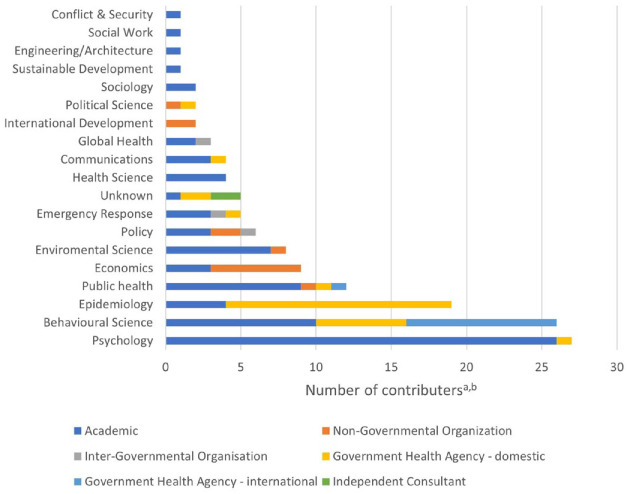
Professional background of contributors ^a^Some individuals work in multiple fields and for multiple organisations; they have been counted once in their primary occupation. ^b^The OECD’s Regulatory Policy Division wrote *OECD 2020* without indicating how many authors there were, so they have been counted once.

Included studies were primarily peer-reviewed journal articles, but there was also a policy paper^19^ and digital educational material.^20^ Eleven records were authored by a multidisciplinary group, bringing a range of professional and academic knowledge to their work (Figure 2).

Four studies were published prior to 2020.^21–24^ Seven records address the response to COVID-19, all published in 2020 and 2021.^5,19,25–29^ Other emergency contexts include flooding,^30^ natural disaster,^22^ chemical incident,^23^ Zika virus,^22^ non-specific infectious disease outbreak,^31,32^ and non-specific emergencies^7,20,24,33^ (Table 1).

**Table 1.** Summary of included records.

	Emergency context	Emergency response type	Behavioural science theories and constructs
Jackson et al.^22^	Natural disasters	Health promotion interventions	Community mobilisation, social marketing
Jalloh et al.^31^	Infectious disease outbreak	Behaviour change implementation approaches	Top-down, intermediary and bottom-up approaches
Lee et al.^25^	COVID-19	Behaviour change interventions	BCW
Morton et al.^7^	Non-specific	Adapting pre-existing interventions	Table of Changes method from the Person-Based Approach
SBCC for Emergency Preparedness^20^	Non-specific	Communication	Socio-ecological model
West et al.^26^	COVID-19	Behaviour change interventions	Various models, but primarily COM-B, BCW and PRIME theory
Kuhlicke et al.^30^	Flooding	Personal protective behaviour	The ‘Behavioural turn’
Michie et al.^27^	COVID-19	Increasing adherence to health protection behaviours	BCW
Oxman et al.^33^	Non-specific	Communication – persuading and informing	N/A
Templeton et al.^28^	COVID-19	Increasing safe behaviour and adherence to guidance	Social identity theory
Carter et al.^23^	Chemical incident	Crowd psychology	Social identity theory
Chater et al.^5^	COVID-19	Rapid guidance development	TRICE, using BCW and COM-B
Organisation for Economic Co-operation and Development (OECD)^19^	COVID-19	Applying behavioural insights to policy	ABCD and BASIC frameworks
Pinchoff et al.^21^	Zika virus	Prioritising interventions	N/A
Linnemayr et al.^24^	Non-specific	Behavioural economics	Invoking social norms, changing the default, framing messages in terms of loss, plan-making and mental mapping, commitment devices, financial micro-incentives, representing small probabilities
Lunn et al.^29^	COVID-19	Increasing adherence to personal protective behaviours	N/A
Weston et al.^32^	Infectious disease outbreak	Behaviour change theories	Health Belief Model, Theory of Planned Behaviour, and Protection Motivation Theory

COM-B: Capability, Opportunity, and Motivation Behaviour model; BCW: Behaviour Change Wheel; TRICE: Template for Rapid Iterative Consensus of Experts; PRIME: Planning, Response, Impulse/Inhibition, Motive, and Evaluation; ABCD: Attention, Belief formation, Choice, and Determination; BASIC: Behavioural, Analysis, Strategy, Intervention, and Change; SBCC: Social and Behaviour Change Communication.

Four records specify a country where their emergency scenarios and response methods occurred.^5,21,27,33^ The UK is the country most represented by the included studies.^5,27,33^

### Purpose and audience

One record aimed to evaluate existing approaches^30^; six proposed new approaches^7,23,24,28,31,33^; and ten described existing approaches.^5,20–22,25–27,29,32^ The emergency response approaches discussed predominantly use behaviour and behaviour change models and theories,^5,20,22–27,30,32^ such as Capability, Opportunity, and Motivation Behaviour model (COM-B) and Planning, Response, Impulse/Inhibition, Motive, and Evaluation theory, as well as implementation,^31^ decision-making,^21,33^ intervention development,^7,19^ and communication theories.^28,29^

Thirteen records specify that their intended audiences are emergency response development and implementation professionals. The remaining four studies do not state an intended audience.^7,25,26,31^ Policymakers and public health officials are the most prevalent of the explicitly mentioned target audiences.

### Behavioural science approaches and theories

The included articles recommend numerous approaches and theories (Table 1). There are multiple uses of the Behaviour Change Wheel,^5,25–27^ COM-B,^5,26^ and social identity theory.^23,28^ Three records do not utilise such approaches,^21,29,33^ and two rely on behavioural science constructs instead.^24,31^

The two records discussing decision-making approaches do not utilise behavioural science theory.^22,33^ The third record that does not use theory is a narrative review of a selection of protective behaviours.^29^

Three records recommend a multidisciplinary approach to behaviour change interventions in an emergency response including stakeholders from various backgrounds to aid in the translation of specialist knowledge into lay terms so that it can be rapidly utilised by emergency response practitioners.^7,22,25^

### Validation of approaches

Four records state that their approach has been tested or validated.^5,23,29,30^ Six records involve established approaches, such as the Behaviour Change Wheel and COM-B, that have been validated previously.^5,22,24–27^ Morton et al.^7^ draw on the Person-Based Approach, which has been validated; however, they do not mention whether their adapted Table of Changes has been tested. Similarly, based on what is said in the article, it is unclear whether the Template for Rapid Iterative Consensus of Experts framework, which draws on the Behaviour Change Wheel and COM-B, has been tested.^5^ Linnemayr et al.^24^ propose behavioural economics methods that have been validated in other fields but not in emergency response. Three of the records that do not mention testing or validation cite a scientific evidence base that forms the basis of their approach,^25,28,32^ suggesting that core concepts have been tested even if the approach itself has not yet. The two records that do not come from academic literature do not state whether they have been tested or validated.^19,20^

### The ACE criteria

This section considers how the content of the included records relates to the ACE criteria outlined in the Introduction section. Five records discuss co-production in developing or implementing an intervention.^7,20,22,25,31^ They do so by recommending the involvement of stakeholders in the intervention design,^7,20^ recommending using community figures to implement bottom-up intervention,^31^ and co-production with the target population.^22,25^ The remaining studies do not mention co-production. There is a range within the included records in the extent to which the approaches consider how to engage and support people from diverse contexts and underserved communities. Five records have this consideration as a part of the approach by recommending participation of local stakeholders to aid in developing tailored interventions and how to gain community support.^23,25,28,29,33^ For example, Jackson et al.^23^ identified that engaging with pre-existing community organisations and community leaders improved the delivery of rapid behavioural science interventions by making communities feel involved in the response. Two records do not have it as part of the approach but do discuss targeted messaging and how to reach isolated communities.^20,24^ Of the records that do not discuss engagement with people from diverse contexts, Michie et al.^27^ mention that equity concerns require further research, and two others discuss models which allow for consideration of diverse or underserved communities.^31,32^ Only three records incorporate co-production with the target population and consider engagement with diverse communities.^20,22,25^

Four records incorporated rapid testing, evaluation, or validation methods for interventions,^5,7,26,27^ two of which used the Acceptability, Practicability, Effectiveness, Affordability, Spill-over effects, and Equity criteria.^26,27^ A further two records discuss testing, evaluation, or validation methods for interventions, but do not mention if it is designed to be used rapidly.^20,25^ Two of the three records that are reviews identify that little of the literature they reviewed included testing, evaluation, or validation of interventions and that future work needs to focus on methodically incorporating this into all interventions.^22,32^

Six records clearly state that their approach is designed to be implemented rapidly.^5,7,19,21,27,29^ Chater et al.^5^ recommend doing so using their template to aid in quicky collating scientific evidence into a single message and translating it into language that can be understood by those implementing it. The remaining records do not state a timeframe for intervention development or implementation using their approach.

**Figure 3:**
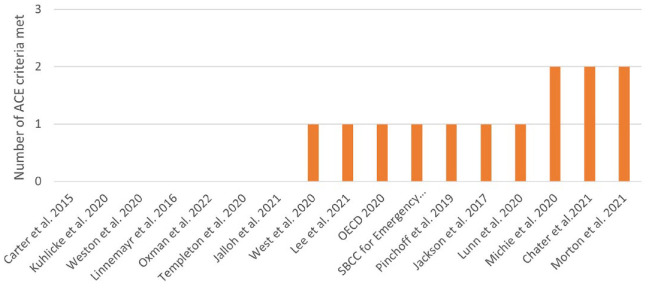
Number of ACE criteria met by the included records Note: ACE: Agile Co-production and Evaluation.

**Figure 4:**
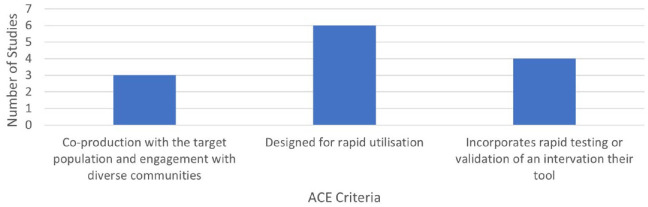
Number of studies to meet each ACE criterion Note: ACE: Agile Co-production and Evaluation.

None of the 17 included records meet all the ACE criteria, and only 3 meet two of the three criteria.^5,7,27^ Seven records meet one criteria,^19–22,25,26,29^ and seven records do not explicitly meet any of the criteria^23,24,28,30–33^ (Figure 3). Three records included co-production with consideration of diversity, six records met the rapid utilisation criterion, and four met the intervention evaluation criterion (Figure 4).

### Summary

Commonalities between the included studies are:

- The challenge of identifying behavioural science knowledge in an emergency context, and the importance of consensus so this knowledge is communicated in a united message.^5,19,21,27^
- The importance of a multidisciplinary approach to behaviour change interventions in emergency response.^7,22,25^
- A lack of robust evaluation of past applications of behavioural science in emergency contexts has left practitioners with a limited evidence base.^7,21,25,26^

## Discussion

Overall, the findings from this review identified that there are relatively few accessible approaches designed to facilitate the use and evaluation of behavioural science in intervention and guidance development during emergency responses. Furthermore, there is a need for a multidisciplinary approach to the development and implementation of behaviour change within emergency response. Given the increased emphasis on behavioural science in emergency response (exemplified by the increase in frameworks and approaches identified herein that were developed post 2020), it is clearly more important than ever to have flexible approaches designed to facilitate the incorporation of behavioural science within incident response.

Of the approaches that do exist, none of them incorporate all the components of the recently developed ACE framework.^13^ The ACE framework was devised in recognition of the need for a unified framework that incorporates rapid implementation, co-production with the target population including seldom heard voices from diverse communities, and evaluation of interventions and messages. Our results support the need for just such a unified framework, as none of the resources included in this review incorporated all the a priori-identified ACE criteria, and existence of this gap, with 10 of the included studies meeting only one or even none.

When considering the broader literature, there is clear evidence for the importance of the criteria against which we have judged the approaches identified within this review for effective emergency response. For instance, co-production is valuable in utilising behavioural science in emergency contexts because members of a target community contribute knowledge, skills, and access to networks that can enhance an intervention’s effectiveness.^34–36^ Similarly, the COVID-19 pandemic and consequent widening of health inequalities demonstrate that considering how to engage diverse populations is critical in emergency response, and behavioural science can be vital to understanding how.^34,36,37^ Consideration of diverse, minority, and disproportionately affected populations can identify and overcome barriers to engagement with health services, tackle stigma, increase resilience, and empower communities, all of which are crucial for emergency response and recovery.^34,38–40^

Furthermore, in terms of a theoretical basis for these approaches, the most prevalent theories and models in the literature were the Behaviour Change Wheel, COM-B, and social identity theory. Behaviour Change Wheel and COM-B are appropriate for emergency response because they are designed to be adaptable to various target behaviours and contexts.^25,41^ Social identity theory can be valuable to emergency response as it can enhance the target population’s ability and willingness to adhere to guidance.^23,28^ The most commonly used approaches to behavioural science therefore seem appropriate to the emergency response context.

In line with the ACE framework, we therefore recommend that future approaches cover theory-driven and evidence-based rapid implementation, co-production with the target population, consideration of engaging under-served communities, and rapid evaluation of interventions. Specific attention should be given to covering all the criteria together to maximise the potential impact of future interventions, messaging, and guidance during an emergency.

Finally, it is also worth noting that the approaches identified within this review are generalised in whom they are intended to be used by. No records are explicitly directed towards specific organisations; however, they all appear to relate to the central management of an emergency; this implies that they were written with central government in mind. The ways central and local governments and external stakeholders can utilise behavioural science differ, as is demonstrated by the separate guidance documents for use in non-emergency situations.^4,12^ It is vital for future emergency preparedness that stakeholders can access behavioural science guidance that addresses their capacities and opportunities for implementation, as the UK government’s *Emergency response and recovery* guidance demonstrates by breaking down the roles of different stakeholders.^42^ Therefore, we recommend that the intended audiences and the needs of said audience be carefully considered when developing such frameworks and toolkits in future.

### Strengths

We are confident in our findings’ internal validity due to the research’s methodological strengths – we prepared a review protocol, searched multiple databases to reduce selection bias, captured grey and published literature, and screened records in duplicate to minimise human error.

### Limitations

Resource limitations meant we could not include potentially relevant hand-identified records in our synthesis or perform data extraction in duplicate.

We have included a list of some of the hand-identified records in Supplemental Material 2, as they may add valuable insight for future work in this topic area. These records were primarily identified after the literature searches had been conducted.

## Conclusions

Behavioural science has significant potential in public health emergency responses. However, it is challenging to implement rigorously and rapidly, which makes having pre-existing approaches to guide rapid implementation critical. The results from this review identified relatively few accessible tools designed to facilitate the use of behavioural science during emergency response. Of those that do exist, none incorporate all the components of the recently developed ACE framework.^13^

Future efforts to incorporate behavioural science into intervention and guidance development during emergency responses should therefore incorporate all elements of the ACE framework. This recommendation is in line with the aims of the ACE framework to produce an effective evidence-based approach for utilising behavioural science in response to a public health emergency.

## Supplemental Material

sj-docx-1-rsh-10.1177_17579139241257102 – Supplemental material for A scoping review of behavioural science approaches and frameworks for health protection and emergency response Supplemental material, sj-docx-1-rsh-10.1177_17579139241257102 for A scoping review of behavioural science approaches and frameworks for health protection and emergency response by Alice Zelenka Martin, D Weston, J M Kesten and C E French in Perspectives in Public Health

sj-docx-2-rsh-10.1177_17579139241257102 – Supplemental material for A scoping review of behavioural science approaches and frameworks for health protection and emergency response Supplemental material, sj-docx-2-rsh-10.1177_17579139241257102 for A scoping review of behavioural science approaches and frameworks for health protection and emergency response by Alice Zelenka Martin, D Weston, J M Kesten and C E French in Perspectives in Public Health
