# Supplementary material for: A scoping review of behavioural science approaches and frameworks for health protection and emergency response
Source: Perspect Public Health. 2024 Jun 10;146(2):95–103. doi: 10.1177/17579139241257102 (PMC13091920; doi:10.1177/17579139241257102)
Supplement: sj-docx-1-rsh-10.1177_17579139241257102 – Supplemental material for A scoping review of behavioural science approaches and frameworks for health protection and emergency response [file sj-docx-1-rsh-10.1177_17579139241257102.docx]

**Supplementary Material 2: Hand-identified relevant records**

1. Bonell C, Michie S, Reicher S, West R, Bear L, Yardley L, et al. Harnessing behavioural science in public health campaigns to maintain ‘social distancing’ in response to the COVID-19 pandemic: key principles. J Epidemiol Community Health. 2020 74(8):617-9.
2. British Psychological Society. Behavioural science and disease prevention: Psychological guidance. 2020. Available from: <https://www.bps.org.uk/sites/www.bps.org.uk/files/Policy/Policy%20-%20Files/Behavioural%20science%20and%20disease%20prevention%20-%20Psychological%20guidance%20for%20optimising%20policies%20and%20communication.pdf>
3. Corker E, Altieri E, Michie S. Enabling countries to apply behavioural science in using global survey data to inform their Covid-19 policies. Qeios. 2021.
4. Collective Service. COVID-19 Behaviour Change Framework. 2021. Available from: <https://www.rcce-collective.net/wp-content/uploads/2021/03/RCCE-COVID-19-Behaviour-Change-Framework.pdf>.
5. López Gómez A, Dogmanas D, Brunet-Adami N, Bagattini N, Bernardi R. Using behavioural and social sciences to inform public policies during COVID-19, Uruguay. Bull World Health Organ. 2021 99(11):843-4.
6. Michie S. Behavioural strategies for reducing covid-19 transmission in the general population. 2020. Available from: <https://blogs.bmj.com/bmj/2020/03/03/behavioural-strategies-for-reducing-covid-19-transmission-in-the-general-population/>.
7. SAGE. Using behavioural science to help minimise the spread of Covid-19. 2021. Available from: <https://www.independentsage.org/wp-content/uploads/2021/11/12th-November-Behavioural-Science-report.pdf>.
8. Tanis C, Nauta F, Boersma M, van der Steenhoven M, Borsboom D, Blanken T. Practical behavioural solutions to COVID-19: Changing the role of behavioural science in crises. Unpublished. DOI: 10.31234/osf.io/q349k.
9. UCL Centre for Behaviour Change. Responding to COVID-19: contributions from the Centre for Behaviour Change. 2020. Available from: <https://blogs.ucl.ac.uk/cbc-covid/>.
10. World Health Organisation. Communicating risk in public health emergencies: A WHO guideline for emergency risk communication (ERC) policy and practice. 2017. Available from: <https://www.who.int/emergencies/risk-communications>.
11. Williams S, Drury J, Michie S, Stokoe E. Covid-19: What we have learnt from behavioural science during the pandemic so far that can help prepare us for the future. 2021. Available from: <https://www.bmj.com/content/375/bmj.n3028.short>.
